# First trimester prenatal screening biomarkers and gestational diabetes mellitus: A systematic review and meta-analysis

**DOI:** 10.1371/journal.pone.0201319

**Published:** 2018-07-26

**Authors:** Brittney M. Donovan, Nichole L. Nidey, Elizabeth A. Jasper, Jennifer G. Robinson, Wei Bao, Audrey F. Saftlas, Kelli K. Ryckman

**Affiliations:** Department of Epidemiology, University of Iowa College of Public Health, Iowa City, Iowa, United States of America; The University of Texas School of Public Health, UNITED STATES

## Abstract

Biomarkers commonly assessed in prenatal screening have been associated with a number of adverse perinatal and birth outcomes. However, it is not clear whether first trimester measurements of prenatal screening biomarkers are associated with subsequent risk of gestational diabetes mellitus (GDM). We aimed to systematically review and statistically summarize studies assessing the relationship between first trimester prenatal screening biomarker levels and GDM development. We comprehensively searched PubMed/MEDLINE, EMBASE, CINAHL, and Scopus (from inception through January 2018) and manually searched the reference lists of all relevant articles. We included original, published, observational studies examining the association of first trimester pregnancy associated plasma protein-A (PAPP-A) and/or free β-human chorionic gonadotropin (free β-hCG) levels with GDM diagnosis. Mean differences were calculated comparing PAPP-A and free β-hCG multiples of median (MoM) levels between women who developed GDM and those who did not and were subsequently pooled using two-sided random-effects models. Our meta-analysis of 13 studies on PAPP-A and nine studies on free β-hCG indicated that first trimester MoM levels for both biomarkers were lower in women who later developed GDM compared to women who remained normoglycemic throughout pregnancy (MD -0.17; 95% CI -0.24, -0.10; MD -0.04; 95% CI -0.07–0.01). There was no evidence for between-study heterogeneity among studies on free β-hCG (I^2^ = 0%). A high level of between-study heterogeneity was detected among the studies reporting on PAPP-A (I^2^ = 90%), but was reduced after stratifying by geographic location, biomarker assay method, and timing of GDM diagnosis. Our meta-analysis indicates that women who are diagnosed with GDM have lower first trimester levels of both PAPP-A and free β-hCG than women who remain normoglycemic throughout pregnancy. Further assessment of the predictive capacity of these biomarkers within large, diverse populations is needed.

## Introduction

Gestational diabetes mellitus (GDM), characterized by abnormal glucose tolerance with onset or first recognition during pregnancy, is the most common metabolic complication in pregnancy and is associated with substantial maternal and neonatal morbidities [1–3]. The prevalence of GDM in the United States has been estimated to be between 3–7%, with rates varying by racial/ethnic background [4]. Observed increases in GDM prevalence rates over the past decade appear to coincide with rising obesity and type 2 diabetes rates across the world [5]. Among the numerous risk factors identified, prior history of GDM is considered the strongest predictor of GDM [6].

Glucose testing for GDM diagnosis is usually performed between 24–28 weeks’ gestation, [6, 7] when maternal insulin resistance increases to preserve nutrients for the rapidly growing fetus [8]. However, evidence of the association between elevated first trimester fasting glucose levels, within the nondiabetic range, and increased risk of GDM diagnosis later in pregnancy and adverse pregnancy outcomes indicate that women with GDM may exhibit metabolic alterations earlier in pregnancy [9–11]. Measurement of first trimester biomarkers representative of these metabolic changes may allow for early detection and management of GDM, improved understanding of GDM pathogenesis, and enhanced targeted intervention [9, 11, 12].

Markers commonly assessed in prenatal screening have been associated with a number of adverse perinatal and birth outcomes when measured at abnormal levels [13–16]. In the absence of aneuploidy and structural anomalies, reduced levels of first trimester pregnancy associated plasma protein-A (PAPP-A) and free β-human chorionic gonadotropin (free β-hCG) are risk factors for preterm delivery, preeclampsia, and spontaneous miscarriage [17]. An inverse relationship between hemoglobin A1c (a marker used to measure glucose maintenance over a three-month time-span) and PAPP-A suggests that PAPP-A may be reflective of the degree of glycemic control within an individual [18, 19]. Because of their potential role in placental pathology and carbohydrate homeostasis, PAPP-A and free β-hCG measurements could be of value in screening for GDM in addition to screening for chromosomal abnormalities [15].

Although numerous studies have investigated the relationship between first trimester PAPP-A and free β-hCG levels and GDM development, results have been conflicting. There is still significant debate as to whether first trimester measurements of prenatal screening biomarkers differ in women with and without GDM and whether these early patterns could aid in GDM prediction. This study aims to systematically review and statistically summarize observational studies assessing the relationship between first trimester prenatal screening biomarker levels and GDM development.

## Methods

This meta-analysis was conducted in adherence with the Meta-analysis Of Observational Studies in Epidemiology (MOOSE) and Preferred Reporting Items for Systematic Reviews and Meta-Analyses (PRISMA) criteria (S1 and S2 Tables) [20].

### Search strategy

A systematic literature search of eligible studies was conducted using PubMed/MEDLINE, EMBASE, CINAHL, and Scopus databases from their inception through January 2018 for studies evaluating the association between first trimester prenatal screening biomarker levels (PAPP-A and/or free β-hCG) and GDM development. A comprehensive search strategy was built with consultation from a research librarian, and included both broad and specific terms describing first trimester biomarkers and GDM. Detailed search strategies for each of the databases searched are listed in the S1 Appendix. To maximize the coverage of our literature search, language restriction on the articles reviewed were not imposed and no filters or limits were placed on the searches. If needed, Google Translate was used to translate articles to English.

The grey literature, or literature that has not been formally published in books or journal articles (e.g., conference abstracts, newsletters, magazines, etc), [21] was not examined for this meta-analysis. While the use of grey literature is thought to decrease the potential for publication bias, it may also induce bias through the inclusion of data that failed to be published because of its low quality [22, 23]. Additional references not detected by the electronic search were identified by manually searching the reference lists of all extracted full-text articles.

### Study selection

Articles were eligible for inclusion in this meta-analysis if they meet the following criteria: 1) an original, published, observational study that examined the association between first trimester PAPP-A and/or free β-hCG levels and diagnosis of GDM, 2) presented multiples of median (MoM) converted PAPP-A and/or free β-hCG measurements as a group mean or median with either a standard deviation (SD), interquartile range (IQR), or 95% confidence interval (CI), 3) included a comparison group of women who didn’t develop GDM (i.e., normoglycemic pregnancies). Review articles, editorials, and non-human studies (i.e., cell culture or animal studies) were excluded. Articles were also excluded if GDM was combined with impaired glucose tolerance or previous cases of type 1 or type 2 diabetes.

We excluded articles in which raw PAPP-A and/or free β-hCG levels were not converted to MoM values (n = 5). In screening using maternal serum biomarkers, raw measurements are often converted into MoM values by dividing each individual’s biomarker level by the median level of that biomarker for the patient population at the same gestational age within the same laboratory. This adjusts for the difference in biomarker levels by gestational age and allows for consistent interpretation of results across laboratories [24]. Additional adjustments for other patient-related factors with the potential to affect biomarker levels were made according to the laboratory’s protocol [24].

The data needed for meta-analysis (i.e., PAPP-A and/or free β-hCG MoM measurements reported as a group mean or median with either a SD, IQR, or 95% CI) were not reported for 9 of the extracted full-text articles. In an attempt to include these articles in our final analyses, we contacted the listed corresponding author. After the second contact attempt, one author responded but did not have the data needed. Therefore, all 9 studies were excluded.

**Fig 1:**
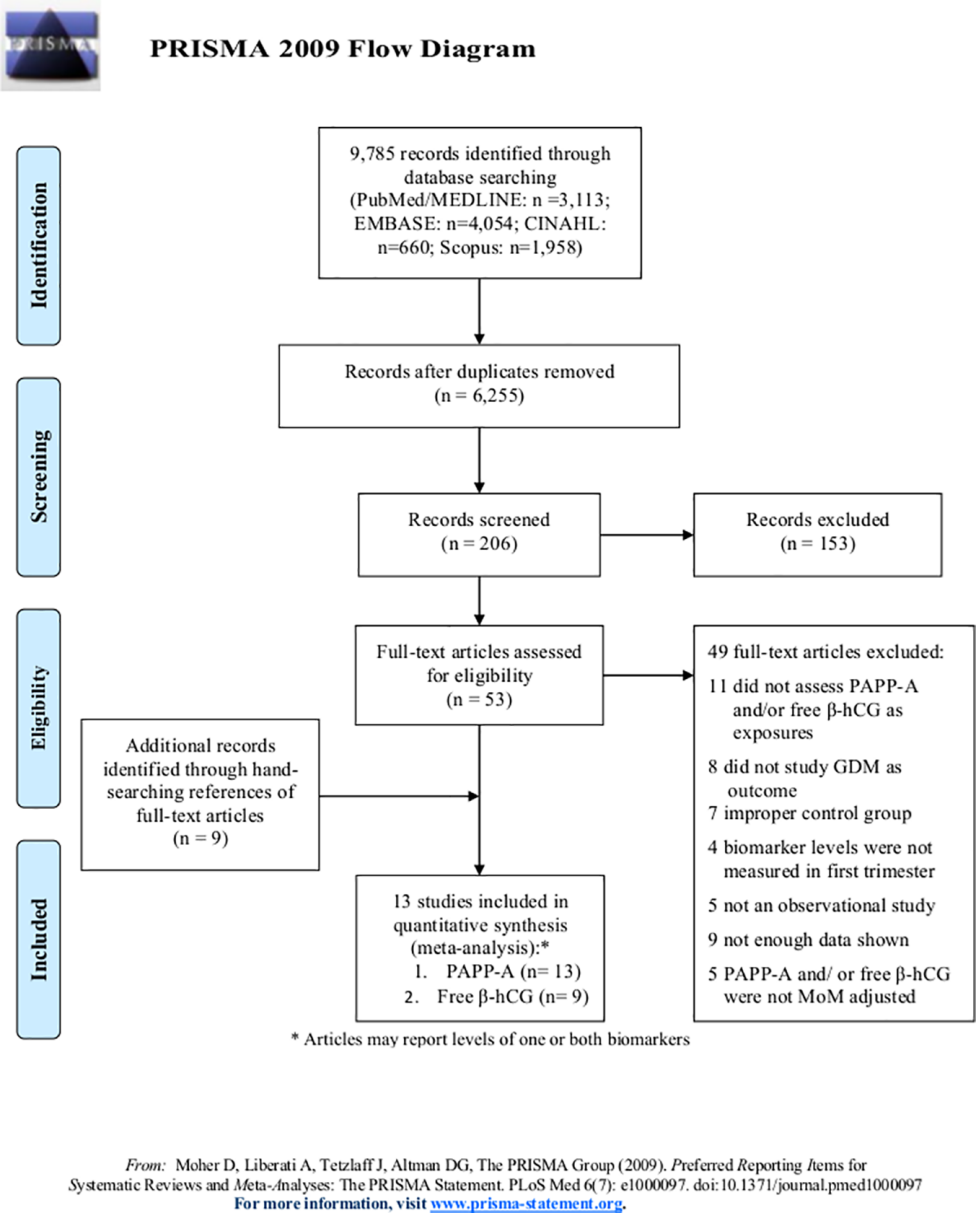
Flow diagram of search strategy.

Articles identified from the literature search were screened for duplicates. All studies deemed to be potentially eligible for inclusion were reviewed and extracted by two independent investigators (BMD and NLN). Results from the search strategy are summarized in Fig 1.

### Data extraction

Full-text articles were obtained for all publications that were deemed eligible for inclusion after title and abstract review. Data was extracted from full-text articles by two independent reviewers (BMD and NLN), using a piloted, customized data extraction form, and included information on study characteristics (author, publication year, journal name, study location, study dates, and study design), participant characteristics (definitions of GDM and control groups and time period and criteria used for GDM diagnosis), and PAPP-A and/or free β-hCG MoM measurements (variables MoM was adjusted for, mean ± SD or median (IQR or 95% CI) of PAPP-A and/or free β-hCG levels among GDM cases and the comparison group, and number of women in each group). The concordance rate for extracted variables was 71% between the investigators, with the majority of differences occurring in quality scoring. Inconsistencies between the two investigators were adjudicated by a third, independent reviewer (EAJ). All investigators had extensive training in the proper conduct and methodology of systematic reviews and meta-analyses prior to the initiation of this meta-analysis.

### Quality assessment

The Newcastle-Ottawa Scale, a quality assessment tool for observational studies, [25] was assessed during data extraction. This tool judges the studies on three broad perspectives: the selection of the study groups, the comparability of the groups, and the ascertainment of either the exposure or the outcome of interest for case-control or cohort studies respectively [25]. Studies were assigned up to nine-stars based on questions within these three perspectives, with more stars indicating higher quality. The scale items were customized to make the questions more applicable to this meta-analysis with regards to defining the control group, adjustment for confounding factors, and ascertainment of the exposure and outcome. Disagreements were resolved by discussion between the two extractors and the adjudicator until a consensus was reached.

### Data synthesis and analysis

First trimester PAPP-A and free β-hCG MoM levels were compared between women who developed GDM and those who did not using mean differences (MDs) and 95% Cis. Before the findings from the included studies could be pooled for meta-analysis, all statistical MoM measurements of PAPP-A and free β-hCG had to be converted into means and SD. Means were approximated from medians, when reported in the full-text article, and SDs were estimated from IQRs and 95% confidence intervals using the following equations [21, 26]:

$$SD\approx \frac{{quartile}_{3}-{quartile}_{1}}{1.35}$$

$$SD\approx \frac{\sqrt{n}\;x\;(upper\;limit-lower\;limit)}{3.92}$$

A main analysis is presented for each biomarker, including the pooled results of all studies assessing that biomarker. To investigate potential sources of between-study heterogeneity, we decided *a priori* to stratify the included studies for each biomarker by geographic location and biomarker assay method. To evaluate the robustness of the pooled estimates, we performed two sensitivity analyses. Pooled estimates were compared before and after the removal of 1) lower quality studies (≤5 stars) and 2) studies with large sample sizes (n >10,000) to assess if larger studies had major influence on the pooled estimates. All analyses were performed using two-sided, random-effects models based on the inverse variance method, which are more conservative in their estimation of the effects than fixed-effects models, allowing for differences in effect-size between studies [1, 27].

Between-study heterogeneity was assessed using the I^2^ statistic. I^2^ values of <25%, 25–75%, and >75% were indicative of low, moderate, and high between-study heterogeneity, respectively [28, 29]. Publication bias was evaluated using funnel plots, and forest plots were provided for visualization of the overall effect size [28]. An Egger’s test for additional assessment of publication bias could not be performed due to the small number of included studies, which decreases the power of this method to detect bias [30]. All statistical analyses were performed using RevMan 5.3 [31].

## Results

### Literature search

Our initial search of the literature yielded a total of 9,785 citations from PubMed/MEDLINE, EMBASE, CINAHL, and Scopus (Fig 1). After removal of duplicates, titles and abstracts of the remaining citations were thoroughly assessed for potential inclusion based on our set criteria. The remaining 53 articles underwent full-text data extraction. Nine additional references (not detected by the electronic search) were identified from hand-searching the reference lists of the full-text articles and were assessed for potential inclusion. After review of the full-text articles, 49 articles were excluded with the most common reason for exclusion being that the study didn’t assess PAPP-A and/or free β-hCG as exposures (n = 11). Our final analysis included 13 articles, nine of which reported first trimester MoM levels of both PAPP-A and free β-hCG, [9, 11, 13, 32–37] four reporting PAPP-A MoM levels only, [38–41] and none reporting only free β-hCG MoM levels.

### Study characteristics

The nine included studies reporting on the association between both first trimester MoM levels of PAPP-A and free β-hCG with GDM development examined a total of 83,921 participants, of which 3,786 (4.5%) were diagnosed with GDM. The four studies reporting solely on the association between PAPP-A MoM levels and GDM diagnosis, not including free β-hCG MoM levels, included 1,275 women with GDM compared to 31,892 normoglycemic controls. Study characteristics are outlined in Table 1. Sample sizes ranged from 72 to 41,786, with the majority of the studies employing a case-control design (three matched and six with unmatched controls). Among the 13 included studies, nine were conducted in Europe, two in Australia, and two in China.

**Table 1:** Characteristics of studies included in the meta-analysis of first trimester prenatal screening biomarker levels and GDM development, 1950-January 2018.

First author, year	Location		Study design	Number of women (cases/controls)	Biomarker measurement			PAPP-A concentration (MoM adjusted mU/L)		Free β-hCG concentration (MoM adjusted ng/mL)		GDM diagnosis	
					Time of blood collection	Assay method	Variables used in MoM adjustment	GDM	Control	GDM	Control	Time of diagnosis	Diagnostic criteria
Beneventi, 2011[32]	Italy		Case-Control	228/228	First trimester	Delfia Xpress	Maternal weight, days of gestation, and whether or not the patient was a smoker	Median (IQR): 0.7 (0.5–1.2)	Median (IQR): 1.2 (0.8–1.6)	Median (IQR): 0.9 (0.6–1.6)	Median (IQR): 1.0 (0.7–1.5)	24–28 weeks gestation	Carpenter and Coustan, 1982; Metzger and Coustan, 1998
Beneventi, 2014[38]	Italy		Case-Control	112/112	First trimester	Delfia Xpress	Not stated	Mean ± SD: 1.06 ± 0.59	Mean ± SD: 1.22 ± 0.64	N/A	N/A	24–28 weeks gestation	Not stated
Cheuk, 2016[11]^a^	China		Case-Control	Early and Late GDM Combined: 169 Early GDM: 43 Control:351	First trimester	Delfia Xpress	Maternal weight and ethnicity	Median (IQR): Combined GDM: 0.97 (0.65–1.32); Early GDM: 0.86 (0.57–1.23)	Median (IQR): 0.99 (0.67–1.44)	Median (IQR): Combined GDM: 1.05 (0.73–1.64); Early GDM: N/A	Median (IQR): 1.02 (0.71–1.55)	Low risk women and those with normal early glucose screening underwent glucose screening ~28–30 weeks gestation	WHO 1999
Farina, 2017[39]	Italy		Matched Case-Control	12/60	First trimester	BRAHMS Kryptor	Gestational age, maternal weight, race, and smoking history	Median (IQR): 0.70 (0.55–1.04)-	Median (IQR): 1.10 (0.72–1.44)	N/A	N/A	24–29 weeks gestation	Not stated
Husslein, 2012[33]	Austria		Matched Case-Control	72/216	11–14 weeks’ gestation	BRAHMS Kryptor	Not stated	Mean ± SD: 1.17 (0.71)	Mean ± SD: 1.13 (0.58)	Mean ± SD: 1.13 (0.73)	Mean ± SD: 1.15 (0.64)	24–28 weeks gestation	German *Diabetes* Society 1993
Kulaksizoglu, 2013[13]	Turkey		Case-Control	60/60	11–14 weeks’ gestation	Immulite 2000	Not stated	Mean ± SD: 0.77 ± 0.42	Mean ± SD: 0.97 ± 0.40	Mean ± SD: 0.93 ± 0.53	Mean ± SD: 0.97 ± 0.29	24–28 weeks gestation	ADA 2003
Lovati, 2013[9]	Italy		Case-Control	307/366	First trimester	Delfia Xpress	Maternal weight, days of gestation, and smoking habit	Mean ± SD: 0.9 ± 0.6	Mean ± SD: 1.3 ± 0.6	Median (IQR): 1 (0.7–1.6)	Median (IQR): 1.05 (0.7–1.6)	24–28 weeks gestation	Before March 2010: IADPSG 2010; After March 2010: ADA 2010
Ong, 2000[34]	England		Cohort	49/4297	73–97 gestational days (10–14 weeks)	BRAHMS Kryptor	Not stated	Median (95% CI): 0.848 (0.691, 1.006)	Median (95% CI): 1.049 (1.028, 1.070)	Median (95% CI): 0.783 (0.587, 0.979)	Median (95% CI): 1.010 (0.984, 1.036)	Harold Wood Hospital: ~24 weeks gestation; King’s College Hospital: 28 weeks gestation	WHO 1980
Savvidou, 2012[35]	England		Cohort	779/41,007	11^+0^–13^+6^ weeks’ gestation	Delfia Xpress	Fetal crown-rump length, maternal weight, smoking, parity, racial origin, and method of conception	Median (IQR): 0.94 (0.65–1.39)	Median (IQR): 1.00 (0.68–1.42)	Median (IQR): 0.95 (0.64–1.51)	Median (IQR): 1.00 (0.68–1.52)	24–28 weeks gestation	WHO
Sweeting, 2017[36]^b^	Australia		Matched Case-Control	Early and Late GDM Combined: 248 Early GDM: 89 Late GDM: 138 Control:732	11^+0^–13^+6^ weeks’ gestation	Immlite 2000	Gestational age, maternal weight, ethnicity, parity, age, smoking, and method of conception	Median (IQR): Combined GDM: 0.81 (0.58–1.20); Early GDM: 0.79 (0.61–1.17); Late GDM: 0.83 (0.57–1.25)	Median (IQR): 1.00 (0.70–1.46)	Median (IQR): Combined GDM: 0.98 (0.64–1.45); Early GDM: 1.07 (0.63–1.50); Late GDM: 0.95 (0.66–1.47)	Median (IQR): 0.99 (0.68–1.55)	Universal testing at 24–28 weeks gestation	ADIPS 1998
Syngelaki, 2015[40]	England		Cohort	787/30,438	11^+0^–13^+6^ weeks’ gestation	Delfia Xpress	Not stated	Median (95% CI): 0.949 (0.913, 0.987)	Median (95% CI): 1.000 (0.994, 1.006)	N/A	N/A	24–28 weeks gestation	WHO 1999
Wells, 2015[41]	Australia	Cohort		Early GDM: 63 Late GDM: 301 Control: 1,282	10–14 weeks’ gestation	Immulite 2000	Maternal weight, ethnicity, and smoking status	Median (IQR): Early GDM: 0.94 (0.63–1.31); Late GDM: 0.79 (0.51–1.28)	Median (IQR): 1.00 (0.68–1.40)	N/A	N/A	Early GDM: first antenatal clinic appointment (15.5 ± 1.5 weeks gestation); Late GDM: 26–28 weeks gestation	ADIPS 1998
Xiao, 2018[37]	China	Case-Control		599/986	11^+0^–13^+6^ weeks’ gestation	Delfia Xpress	Not stated	Median (IQR): 0.88 (0.60–1.28)	Median (IQR): 0.97 (0.67–1.37)	Median (IQR): 1.01 (0.69–1.58)	Median (IQR): 1.06 (0.73–1.62)	Late second trimester	IADPSG 2010

WHO, World Health Organization; ADA, American Diabetes Association; IADPSG, International Association of Diabetes and Pregnancy Study Groups; ADIPS, Australasian Diabetes in Pregnancy Society

^a^Women with ≥1 risk factors for the development of GDM underwent early glucose testing and could have potentially been diagnosed after the initial prenatal visit. PAPP-A and free β-hCG MoM measurements were reported for the combined GDM group (including those diagnosed early and later pregnancy) and for those who were diagnosed with GDM early in pregnancy. Separate PAPP-A and free β-hCG MoM measurements weren’t reported for those women who were diagnosed late in pregnancy.

^b^Cases were defined as women who had a diagnosis of GDM made at any time point during pregnancy. GDM was classified as either “early GDM” or “standard GDM” depending on whether diagnosis occurred < or ≥24 weeks’ gestation. PAPP-A and free β-hCG MoM measurements were given for the combined GDM group (including those diagnosed early and later in pregnancy and those without data on the time of diagnosis) and separately for those who diagnosed early and later in pregnancy.

Studies used one of three primary assay methods to measure PAPP-A and free β-hCG MoM levels: Delfia Xpress (Perkin-Elmer, Waltham, MA, USA) (n = 7), BRAHMS Kryptor (Thermo Fisher Scientific Inc., Brahms GmbH, Henningsdorf, Germany) (n = 3), and Immulite 2000® (Diagnostic Products Corp., Los Angeles, CA, USA) (n = 3). As per our inclusion criteria, all studies collected blood for biomarker MoM measurements within the first trimester of pregnancy (≤14 weeks’ gestation). MoM values were adjusted for additional patient-related variables in six of the included studies. Common variables used in this adjustment included maternal weight, smoking history, and race/ethnicity. Diagnosis of GDM was usually made between 24–28 weeks gestation. The criteria used to diagnose GDM varied across the studies, including criteria established by Carpenter and Coustan, the German Diabetes Society, the World Health Organization, the American Diabetes Association, the Australasian Diabetes in Pregnancy Society, and the International Association of Diabetes and Pregnancy Study Groups (S3 Table). Mean MoM levels of GDM women ranged from 0.70–1.17 for PAPP-A and 0.78–1.13 for free β-hCG and 0.97–1.22 and 0.97–1.15 for PAPP-A and free β-hCG in normoglycemic women, respectively. The included studies were of similar quality, with the majority of the studies scoring ≥7 stars (out of nine stars) [9, 11, 32, 35–38]. Of the six studies scoring less than seven stars, only one received a score less than five [13].

In three studies, [11, 36, 41] early-pregnancy selective screening was utilized in addition to universal screening later in pregnancy to identify women of high risk for developing GDM (such as those with a family history of diabetes, high BMI, or previous GDM) earlier in pregnancy. Based on their glucose test results, these women could have been diagnosed with GDM before routine universal testing at 24–28 weeks’ gestation was employed. To determine if the MDs for first trimester PAPP-A and free β -hCG differed depending on timing of GDM diagnosis, we conducted a *post-hoc* stratified analysis in which pooled MDs were calculated separately for studies reporting biomarker MoM measurements for women who were diagnosed with GDM early and later in pregnancy. Sweeting et al. [36] and Wells et al. [41] reported separate PAPP-A MoM measurements for early and late GDM diagnostic groups. For these studies, pooled MDs were calculated for PAPP-A MoM levels reported for those diagnosed with GDM early in pregnancy and the PAPP-A MoM measurements for those diagnosed with GDM later in pregnancy were pooled with all other studies for each biomarker. The other study, Cheuk et al., [11] only reported separate PAPP-A MoM levels for the combined GDM group (including those diagnosed early and late in pregnancy) and the early GDM diagnostic group. Therefore, this study was included in the pooled MD calculation for early GDM diagnosis but was removed from the pooled estimate for late GDM diagnosis.

Because Sweeting et al. [36] was the only study to report separate free β-hCG MoM levels for women who were diagnosed with GDM early and later in pregnancy, we were unable to perform a stratified analysis to determine if free β-hCG differed depending on timing of GDM diagnosis.

### Meta-analysis of PAPP-A

**Fig 2:**
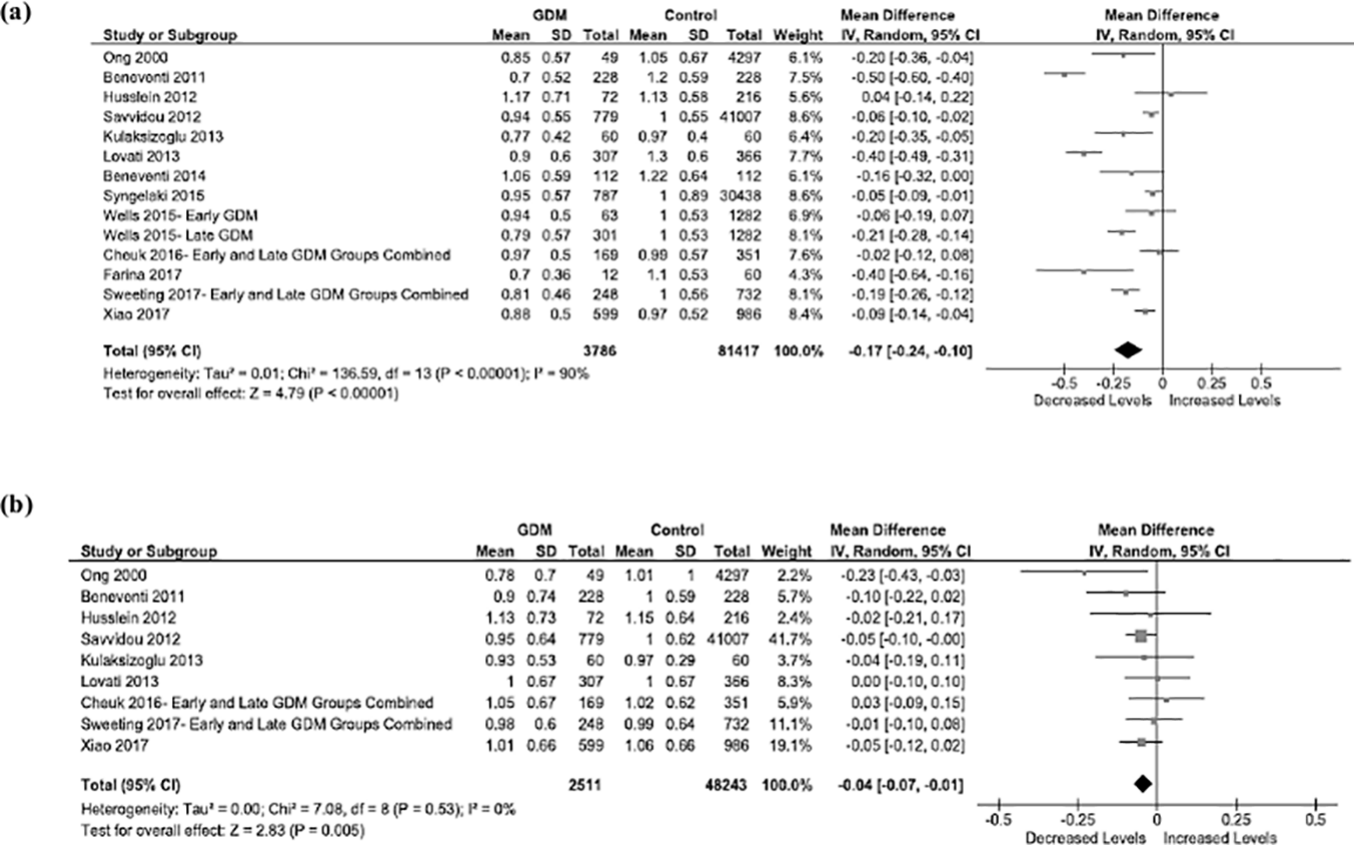
Forest plots of all studies reporting on first trimester (a) PAPP-A MoM levels and (b) free β-hCG MoM levels for women with and without gestational diabetes mellitus.

Thirteen studies reported on the association between first trimester PAPP-A MoM levels and GDM development. Results from the meta-analysis showed that first trimester PAPP-A MoM levels were significantly lower in women who later developed GDM compared to women who remained normoglycemic throughout pregnancy (random-effects pooled MD -0.17; 95% CI -0.24, -0.10; p <0.00001) (Fig 2(A)). Symmetry of the studies within the funnel plot indicated minimal publication bias (S1 Fig). A high level of between-study heterogeneity was detected among the studies reporting PAPP-A levels (I^2^ = 90%).

To assess potential sources of heterogeneity, studies were stratified by geographic location (Europe, Australia, and Asia) and biomarker assay method (Delfia Xpress, BRAHMS Kryptor, and Immulite 2000). First trimester PAPP-A MoM mean levels were not as low among women who later developed GDM in the two studies performed in Asia compared to the studies performed in Europe (n = 9) or Australia (n = 2) (MD -0.07; 95% CI -0.13, -0.00 vs. MD -0.21; 95% CI -0.33, -0.10 and MD -0.17; 95% CI -0.24, -0.10; p = 0.03) (S2 Fig). MDs in PAPP-A MoM levels did not differ when stratified by biomarker assay method (S3 Fig). Between-study heterogeneity was attenuated in studies performed in Australia and Asia (I^2^ = 52% and 37%) but not in studies performed in Europe (I^2^ = 93%). Between-study heterogeneity was also attenuated in studies using BRAHMS Kryptor and Immulite 2000 (I^2^ = 77% and 30%) but not in studies using the Delfia Xpress method (I^2^ = 95%).

Sensitivity analyses were performed to evaluate the influence of sample size and study quality on the pooled MDs reporting PAPP-A MoM levels. The overall effect was slightly more pronounced after exclusion of two large cohort studies [35, 40] (MD -0.20; 95% CI -0.28, -0.11) (S4 Fig). First trimester PAPP-A MoM levels were still significantly lower among women who later developed GDM compared to controls when one study of lower quality [13] was removed (MD -0.17; 95% CI -0.25, -0.10) (S5 Fig). The between-study heterogeneity decreased only slightly after removal of large and lower quality studies (I^2^ = 89% and 91%).

An additional *post-hoc* stratified analysis was done to determine if PAPP-A levels differed depending on timing of GDM diagnosis. The pooled MD for PAPP-A was lower, although not significantly, for women who were diagnosed with GDM later in pregnancy (24–28 weeks gestation) than women who were diagnosed early in pregnancy (<24 weeks gestation) compared to normoglycemic women (MD -0.19; 95% CI -0.27, -0.11 vs. MD -0.14; 95% CI -0.24, -0.05; p = 0.41) (S6 Fig). The heterogeneity among studies reporting first trimester PAPP-A MoM levels for women diagnosed with GDM early in pregnancy was moderate (I^2^ = 44%).

### Meta-analysis of free β-hCG

The association between first trimester free β-hCG MoM levels and GDM development was estimated using data from nine studies. The pooled MD showed lower MoM levels of first trimester free β-hCG among women who later developed GDM compared to women who remained normoglycemic throughout pregnancy (random-effects pooled MD -0.04; 95% CI -0.07, -0.01; p = 0.005) (Fig 2(B)). Funnel plot symmetry suggested no publication bias (S7 Fig). The studies reporting free β-hCG MoM levels appeared to be homogenous in their findings (I^2^ = 0%). Therefore, no subgroup analyses were performed [42].

Similar to what was observed in the sensitivity analyses for studies reporting on first trimester PAPP-A MoM measurements, the pooled MD and I^2^ for free β-hCG MoM measurements remained unchanged after exclusion of a large cohort study [35] (MD -0.04; 95% CI -0.08, 0.00; I^2^ = 0%) and a lower-quality study [13] (MD -0.04; 95% CI -0.07, -0.01; I^2^ = 1%) (S8 and S9 Figs). Because only one study reported separate free β-hCG MoM levels for women who were diagnosed with GDM early and later in pregnancy, we were unable to perform a stratified analysis to determine if free β-hCG differed depending on timing of GDM diagnosis.

## Discussion

Our meta-analysis indicates that women diagnosed with GDM have lower first trimester levels of both PAPP-A and free β-hCG than women who remain normoglycemic throughout pregnancy. There was no evidence of heterogeneity among the first trimester free β-hCG MoM studies. However, between-study heterogeneity was detected for studies reporting first trimester PAPP-A MoM levels; heterogeneity was reduced after stratifying by geographic location and biomarker assay method, suggesting that these factors account for some of the inherent differences among the studies for first trimester PAPP-A. Overall, first trimester PAPP-A MoM levels were lower in women diagnosed with GDM than normoglycemic women; however, this effect was slightly smaller among studies performed in Asia compared to studies performed in Europe or Australia. Women of Asian descent have been shown to have higher PAPP-A levels compared to Caucasian women, [43] and our findings suggest that this may attenuate the association between PAPP-A levels and progression to GDM. However, only two studies included in this meta-analysis were conducted within primarily Asian populations warranting further examination of this relationship within ethnically diverse populations.

Results from our sensitivity analyses indicated that our findings for both PAPP-A and free β-hCG were robust to the assumption of study quality and size. Because the majority of the studies included in this meta-analysis were found to be of higher quality, the risk of bias was low. Through *post-hoc* stratified analysis, we found that PAPP-A MoM levels were reduced at nearly the same extent for women diagnosed with GDM earlier in pregnancy, through selective screening based on risk factors, compared to those who were diagnosed later in pregnancy, through universal screening. This illustrates that detection of lower PAPP-A levels early in pregnancy may be a useful indicator of GDM risk in women without traditional risk factors (e.g., family history of diabetes, high BMI, etc.).

### Strengths and limitations

A major strength of this meta-analysis was the use of a thorough search strategy developed with the aid of a research librarian with extensive training in the methods of systematic reviews. By conducting our search within four large databases (PubMed/MEDLINE, EMBASE, CINAHL, and Scopus) and hand-searching the reference lists of the full-text articles to detect references that may have been missed through electronic searches, we feel confident that all relevant articles have been identified. The potential for language bias was limited by allowing articles of all languages to be assessed for inclusion. Although the final set of articles included in this meta-analysis were all written in English, several articles were partially translated to assess for inclusion. Ancillary efforts were made to contact authors when additional data was needed for study inclusion. Although the grey literature wasn’t examined for this meta-analysis, the symmetry observed within our funnel plots suggests that publication bias wasn’t of concern.

Limitations of this meta-analysis should be noted. A large amount of between-study heterogeneity was observed when pooling together studies reporting on first trimester PAPP-A MoM measurements, which was reduced, but not totally accounted for, when stratifying by geographic location and biomarker assay method. Lack of consistency in the criteria used in GDM diagnosis may have contributed to this; however, because so few studies used the same criteria, we were unable to perform an additional stratified analysis using this factor. The influence of study design on between-study heterogeneity was assessed in a *post-hoc* analysis. Heterogeneity remained high when stratifying studies reporting on first trimester PAPP-A MoM measurements by study design (cohort studies: I^2^ = 78%, case-control studies: I^2^ = 91%).

As methods describing the collection, storage, and handling of blood samples were sparse within the included studies, errors in the measurement of biomarkers may have biased our pooled estimates. Bias in the association between first trimester prenatal screening markers and GDM development may have also been introduced through the use of different variables for adjusting the MoM values within different laboratories [11]. We included studies that reported either median or mean MoM values, using median values to approximate means as recommended by the Cochrane Collaboration for continuous outcomes and required by the RevMan analysis software [21, 31]. As medians are the preferred statistical measure of first trimester prenatal screening biomarker levels, these values may not completely approximate the median values. While mean differences in biomarkers levels between women who developed GDM and those who did not were the primary focus of this meta-analysis, characteristics of a biomarker threshold that could be used in clinical practice were not determined. Threshold determinations, along with assessment of other biomarkers with potentially higher predictive power for detection of GDM, should be the focus of future studies.

Women with pre-existing diabetes may have been misclassified as having GDM through the use of universal screening in mid- to late-pregnancy in the included studies. It has been suggested that the reductions in PAPP-A are proportional to the severity of maternal hyperglycemia, with levels decreasing from late-onset GDM, early-onset GDM, to pre-existing type 2 diabetes [41]. Although our *post-hoc* analysis showed that the pooled MD for PAPP-A MoMs were similar between studies reporting PAPP-A MoM levels for women who were diagnosed with GDM early in pregnancy and studies reporting on women who were diagnosed later in pregnancy, the significant reductions in first trimester PAPP-A among women with GDM compared to normoglycemic women suggest that this biomarker may be a useful indicator of the presence of glucose intolerance at the start of pregnancy [32, 37, 41].

### Interpretation

Gestational diabetes is a common metabolic complication of pregnancy that is associated with substantial maternal and neonatal morbidity [15]. The standard of care for GDM in most developed countries is universal mid- to late- pregnancy glucose testing, [6, 7] which leaves limited opportunity for early diagnosis and management. Detection of dysregulation in biomarkers potentially associated with the pathophysiology of GDM could aid in the recognition of those at high risk of GDM earlier in pregnancy [12].

First trimester prenatal screening for fetal aneuploidy is a widely accepted obstetric practice [24, 33]. Aneuploidy risk calculation is based on the measurement of two biochemical markers, pregnancy associated plasma protein-A (PAPP-A) and free β-human chorionic gonadotropin (free β-hCG), from maternal serum [24]. PAPP-A and hCG are glycoprotein hormones produced by the placenta and found in the plasma of pregnant women [24, 44, 45]. The primary function of PAPP-A is to increase the bioavailability of insulin like growth factor, ultimately facilitating glucose and amino acid transport into the placenta [24]. Free β-hCG, one of two subunits of the hCG hormone, exerts growth promoting activity and is associated with an increased risk of Down syndrome when found at increased levels within maternal blood [24]. Because of their role in placental development and carbohydrate homeostasis, these markers could be of value in identifying women early in pregnancy who may needed further follow-up screening for GDM in addition to screening for chromosomal abnormalities [15]. To our knowledge, this is the first meta-analysis exploring the relationship between first trimester prenatal screening biomarker levels and GDM development.

Although the temporal relationship between low PAPP-A and free β-hCG levels and GDM is not fully understood, our findings suggest that the incorporation of early pregnancy maternal serum biomarkers into risk-prediction models could aid in the earlier identification of women at risk for GDM development. Five of the thirteen studies included in the meta-analysis examined the utility of incorporating first trimester prenatal screening markers into GDM risk prediction models. Farina et al., [39] Lovati et al., [9] and Sweeting et al. [36] found that models integrating first trimester biomarkers with maternal characteristics showed increased predictive ability compared with models that included maternal characteristics alone. In contrast, Syngelaki et al. [40] and Xiao et al. [37] found that models including maternal risk factors alone and in combination with PAPP-A measurements didn’t differ in their ability to predict GDM development. The interpretation of the predictive values presented in these studies are of limited value as all but one (Syngelaki et al.) [40] were of case-control design. A case-control design produces a biased sample (i.e., the proportion of cases in the sample is not the same as the population of interest), rendering the risk modeling approach for prediction ineffective [46].

It is important to note that the pooled differences observed in first trimester prenatal screening biomarkers observed in this meta-analysis, while significantly reduced, are small in magnitude and, thus, may not be clinically useful when used alone in prediction algorithms. However, using these markers in combination with other biomarkers of oxidative stress and insulin resistance could prove to be useful in predicting GDM risk early in pregnancy [1]. Further assessment of the predictive capacity of these biomarkers within prospective studies of large, diverse populations is needed. Even a modest increase in the ability to predict GDM is important as these biomarkers are already being measured as part of routine antenatal care and could provide a cost-effective and unified approach to early pregnancy screening for potential complications [36].

## Conclusions

Our findings indicate that women diagnosed with GDM have lower first trimester levels of both PAPP-A and free β-hCG than women who remain normoglycemic throughout pregnancy. These biomarkers may serve as indicators of the presence of abnormal glucose metabolism at the start of pregnancy and could aid in the identification of women at risk for GDM development. Further assessment of the predictive capacity of these biomarkers within large, diverse populations is warranted for effective clinical utility.

## Supporting information

S1 Appendix Database search strategies. (DOCX)

S1 Table MOOSE checklist for meta-analyses of observational studies. (PDF)

S2 Table PRISMA 2009 checklist. (PDF)

S3 Table Commonly used venous plasma glucose concentration diagnostic thresholds for gestational diabetes using an oral glucose tolerance test. (PDF)

S1 Fig Funnel plot of the studies reporting on PAPP-A MoM levels among women with and without GDM. (PDF)

S2 Fig Forest plot of the studies reporting on PAPP-A MoM levels among women with and without GDM stratified by geographic location. (PDF)

S3 Fig Forest plot of the studies reporting on PAPP-A MoM levels among women with and without GDM stratified by biomarker assay method. (PDF)

S4 Fig Forest plot of the studies reporting on PAPP-A MoM levels among women with and without GDM removing large studies. (PDF)

S5 Fig Forest plot of the studies reporting on PAPP-A MoM levels among women with and without GDM removing a study of lower quality. (PDF)

S6 Fig Forest plot of the studies reporting on PAPP-A MoM levels among women with and without GDM stratified by timing of GDM diagnosis. (PDF)

S7 Fig Funnel plot of the studies reporting on free β-hCG MoM levels among women with and without GDM. (PDF)

S8 Fig Forest plot of the studies reporting on free β-hCG MoM levels among women with and without GDM removing a large study. (PDF)

S9 Fig Forest plot of the studies reporting on free β-hCG MoM levels among women with and without GDM removing a study of lower quality. (PDF)
