# Supplementary material for: First trimester prenatal screening biomarkers and gestational diabetes mellitus: A systematic review and meta-analysis
Source: PLoS One. 2018 Jul 26;13(7):e0201319. doi: 10.1371/journal.pone.0201319 (PMC6062092; doi:10.1371/journal.pone.0201319)
Supplement: S1 Appendix — (DOCX) [file pone.0201319.s001.docx]

**S1 Appendix.** Database search strategies.

**PubMed/MEDLINE**

"Pregnancy-Associated Plasma Protein-A"[Mesh] OR Pregnancy-Associated Plasma Protein-A [tw] OR Pregnancy Associated Plasma Protein A [tw] OR IGFBP-4 Protease [tw] OR IGFBP 4 Protease [tw] OR IGFBP-4-Specific Proteinase [tw] OR Insulin-Like Growth Factor-Dependent IGF Binding Protein-4 Protease [tw] OR Insulin Like Growth Factor Dependent IGF Binding Protein 4 Protease [tw] OR PAPP-A [tw] OR PAPP alpha [tw] OR Pregnancy Associated alpha Plasma Protein [tw] OR Pregnancy-Associated alpha-Plasma Protein [tw] OR IGFBP-4 Metalloproteinase [tw] OR IGFBP 4 Metalloproteinase [tw] OR Insulin-Like-Growth Factor Binding Protein-4 Protease [tw] OR Insulin Like Growth Factor Binding Protein 4 Protease [tw] OR "Chorionic Gonadotropin"[Mesh] OR Chorionic Gonadotropin [tw] OR Choriogonadotropin [tw] OR Choriogonin [tw] OR Pregnyl [tw] OR Chorulon [tw] OR Gonabion [tw] OR Human Chorionic Gonadotropin [tw] OR HCG (Human Chorionic Gonadotropin) [tw] OR "Biomarkers"[Mesh] OR Biomarker* [tw] OR Biologic Marker* [tw] OR Biological Marker* [tw] OR Laboratory Marker* [tw] OR Serum Marker* [tw] OR Clinical Marker* [tw] OR Biochemical Marker* [tw] OR "Aneuploidy"[Mesh] OR Aneuploid* [tw] OR Aneuploid Cell* [tw] OR "Prenatal Diagnosis"[Mesh] OR Prenatal Diagnos* [tw] OR Intrauterine Diagnos* [tw] OR Antenatal Diagnos* [tw] OR Prenatal Screening* [tw] OR Antenatal Screening* [tw] OR "Pregnancy Trimester, First"[Mesh] OR First Trimester Pregnanc* [tw] OR First Pregnancy Trimester* [tw] OR Early Placental Phase* [tw] OR First Trimester* [tw] OR early pregnancy [tw]

AND

"Diabetes, Gestational"[Mesh] OR gestational diabetes [tw] OR Pregnancy-Induced Diabetes [tw] OR Gestational Diabetes Mellitus [tw] OR gestational diabetic* [tw] OR diabetic pregnanc* [tw] OR GDM [tw]

**EMBASE**

'pregnancy associated plasma protein a'/exp OR 'papp a':ti,ab OR 'pregnancy associated alpha plasma protein':ti,ab OR 'pregnancy associated plasma protein a':ti,ab OR 'pregnancy-associated alpha-plasma protein':ti,ab OR 'pregnancy-associated plasma protein-a':ti,ab OR 'chorionic gonadotropin beta subunit'/exp OR 'beta hcg':ti,ab OR 'beta human chorionic gonadotrophin':ti,ab OR 'beta human chorionic gonadotropin':ti,ab OR 'beta subunit hcg':ti,ab OR 'choriogonadotropin beta subunit':ti,ab OR 'chorionic gonadotropin beta subunit':ti,ab OR 'hcg beta subunit':ti,ab OR 'human chorionic gonadotropin beta subunit':ti,ab OR 'biological marker'/exp OR 'bioindicator':ti,ab OR 'biological indicator':ti,ab OR 'biological marker*':ti,ab OR 'biomarker':ti,ab OR 'aneuploidy'/exp OR 'aneploidy':ti,ab OR 'aneuploid':ti,ab OR 'aneuploidy':ti,ab OR 'chromosome aneuploidy':ti,ab OR 'prenatal diagnosis'/exp OR 'antenatal diagnosis':ti,ab OR 'prenatal diagnosis':ti,ab OR 'prenatal screening'/exp OR 'prenatal screening':ti,ab OR 'first trimester pregnancy'/exp OR 'early pregnancy':ti,ab OR 'first trimester':ti,ab OR 'first trimester pregnancy':ti,ab

AND

'pregnancy diabetes mellitus'/exp OR 'diabetes mellitus gravidarum':ti,ab OR 'gestational diabetes':ti,ab OR 'gestational diabetes mellitus':ti,ab OR 'pregnancy diabetes':ti,ab OR 'pregnancy diabetes mellitus':ti,ab OR 'pregnancy in diabetics':ti,ab

**CINAHL**

“pregnancy-associated plasma protein-A" OR “PAPP-A” OR “PAPP alpha” OR “Pregnancy Associated alpha Plasma Protein” OR “IGFBP 4 Metalloproteinase” OR “Insulin-Like-Growth Factor Binding Protein-4 Protease” OR “Insulin Like Growth Factor Binding Protein 4 Protease” OR (MH "Gonadotropins, Chorionic") OR “Chorionic Gonadotropin” OR “Choriogonadotropin” OR “Pregnyl” OR “Human Chorionic Gonadotropin” OR “HCG” OR (MH "Biological Markers+") OR “Biomarker*” OR “Biologic Marker*” OR “Biological Marker*” OR “Laboratory Marker*” OR “Serum Marker*” OR “Clinical Marker*” OR “Biochemical Marker*” OR (MH "Aneuploidy") OR “Aneuploid*” OR “Aneuploid Cell*” OR (MH "Prenatal Diagnosis+") OR “Prenatal Diagnos*” OR “Intrauterine Diagnos*” OR “Antenatal Diagnos*” OR “Prenatal Screening*” OR “Antenatal Screening*” OR (MH "Pregnancy Trimester, First") OR “First Trimester Pregnanc*” OR “First Pregnancy Trimester*” OR “Early Placental Phase*” OR “First Trimester*” OR “early pregnancy”

AND

(MH "Diabetes Mellitus, Gestational") OR “gestational diabetes” OR “Pregnancy-Induced Diabetes” OR “Gestational Diabetes Mellitus” OR “gestational diabetic*” OR “diabetic pregnanc*” OR “GDM”

**Scopus**

“Pregnancy-Associated Plasma Protein-A” OR “Pregnancy Associated Plasma Protein A” OR “IGFBP-4 Protease” OR “IGFBP 4 Protease” OR “IGFBP-4-Specific Proteinase” OR “Insulin-Like Growth Factor-Dependent IGF Binding Protein-4 Protease” OR “Insulin Like Growth Factor Dependent IGF Binding Protein 4 Protease” OR “PAPP-A” OR “PAPP alpha” OR “Pregnancy Associated alpha Plasma Protein” OR “Pregnancy-Associated alpha-Plasma Protein” OR “IGFBP-4 Metalloproteinase” OR “IGFBP 4 Metalloproteinase” OR “Insulin-Like-Growth Factor Binding Protein-4 Protease” OR “Insulin Like Growth Factor Binding Protein 4 Protease” OR “Chorionic Gonadotropin” OR “Choriogonadotropin” OR “Choriogonin” OR “Pregnyl” OR “Chorulon” OR “Gonabion” OR “Human Chorionic Gonadotropin” OR “HCG” OR “Biomarker*” OR “Biologic Marker*” OR “Laboratory Marker*” OR “Serum Marker*” OR “Clinical Marker*” OR “Biochemical Marker*” OR “Aneuploid*” OR “Aneuploid Cell*” OR “Prenatal Diagnos*” OR “Intrauterine Diagnos*” OR “Antenatal Diagnos*” OR “Prenatal Screening*” OR “First Trimester Pregnanc*” OR “First Pregnancy Trimester*” OR “Early Placental Phase*” OR “First Trimester*” OR “early pregnancy”

AND

“gestational diabetes” OR “Pregnancy-Induced Diabetes” OR “Gestational Diabetes Mellitus” OR “gestational diabetic*” OR “diabetic pregnanc*” OR “GDM”
