# Supplementary material for: First trimester prenatal screening biomarkers and gestational diabetes mellitus: A systematic review and meta-analysis
Source: PLoS One. 2018 Jul 26;13(7):e0201319. doi: 10.1371/journal.pone.0201319 (PMC6062092; doi:10.1371/journal.pone.0201319)
Supplement: S1 Table — (PDF) [file pone.0201319.s002.pdf]

MOOSE Checklist for Meta-analyses of Observational Studies

Item No	Recommendation	Reported on Page No
Reporting of background should include		
1	Problem definition	3-4
2	Hypothesis statement	4
3	Description of study outcome(s)	3
4	Type of exposure or intervention used	3-4
5	Type of study designs used	4
6	Study population	4
Reporting of search strategy should include		
7	Qualifications of searchers (eg, librarians and investigators)	4, 6
8	Search strategy, including time period included in the synthesis and key words	S1 Appendix & p.4
9	Effort to include all available studies, including contact with authors	4-6
10	Databases and registries searched	4
11	Search software used, name and version, including special features used (eg, explosion)	N/A
12	Use of hand searching (eg, reference lists of obtained articles)	5
13	List of citations located and those excluded, including justification	Fig 1 & p.5-6
14	Method of addressing articles published in languages other than English	4
15	Method of handling abstracts and unpublished studies	5
16	Description of any contact with authors	5-6
Reporting of methods should include		
17	Description of relevance or appropriateness of studies assembled for assessing the hypothesis to be tested	5
18	Rationale for the selection and coding of data (eg, sound clinical principles or convenience)	6
19	Documentation of how data were classified and coded (eg, multiple raters, blinding and interrater reliability)	6
20	Assessment of confounding (eg, comparability of cases and controls in studies where appropriate)	6
21	Assessment of study quality, including blinding of quality assessors, stratification or regression on possible predictors of study results	6-7
22	Assessment of heterogeneity	7-8
23	Description of statistical methods (eg, complete description of fixed or random effects models, justification of whether the chosen models account for predictors of study results, dose-response models, or cumulative meta-analysis) in sufficient detail to be replicated	7-8
24	Provision of appropriate tables and graphics	Tables & Figures
Reporting of results should include		
25	Graphic summarizing individual study estimates and overall estimate	Figure 2
26	Table giving descriptive information for each study included	Table 1
27	Results of sensitivity testing (eg, subgroup analysis)	Supplemental Figures & p.14-16
28	Indication of statistical uncertainty of findings	14-16

Item No	Recommendation	Reported on Page No
Reporting of discussion should include		
29	Quantitative assessment of bias (eg, publication bias)	S1 Fig & S7 Fig
30	Justification for exclusion (eg, exclusion of non-English language citations)	8
31	Assessment of quality of included studies	13
Reporting of conclusions should include		
32	Consideration of alternative explanations for observed results	16-18
33	Generalization of the conclusions (ie, appropriate for the data presented and within the domain of the literature review)	19-20
34	Guidelines for future research	18-20
35	Disclosure of funding source	N/A

From: Stroup DF, Berlin JA, Morton SC, et al, for the Meta-analysis Of Observational Studies in Epidemiology (MOOSE) Group. Meta-analysis of Observational Studies in Epidemiology. A Proposal for Reporting. *JAMA*. 2000;283(15):2008-2012. doi: 10.1001/jama.283.15.2008.

Transcribed from the original paper within the NEUROSURGERY® Editorial Office, Atlanta, GA, United Sates. August 2012.
