# Supplementary material for: First trimester prenatal screening biomarkers and gestational diabetes mellitus: A systematic review and meta-analysis
Source: PLoS One. 2018 Jul 26;13(7):e0201319. doi: 10.1371/journal.pone.0201319 (PMC6062092; doi:10.1371/journal.pone.0201319)
Supplement: S3 Table — (PDF) [file pone.0201319.s004.pdf]

S1 Table. Commonly used venous plasma glucose concentration diagnostic thresholds for gestational diabetes using an oral glucose tolerance test.

Criteria, Year	Approach	Glucose Load (g)	Glucose Threshold (mg/dL (mmol/L))				Number of abnormal values required for diagnosis
			Fasting	1 hour post-glucose load	2 hour post-glucose load	3 hour post-glucose load	
Carpenter and Coustan[1]	2-step*	100	≥95 (5.3)	≥180 (10.0)	≥155 (8.6)	≥140 (7.8)	≥2
German Diabetes Society[2]	1-step	75	≥92 (5.1)	≥180 (10.0)	≥153 (8.5)	Not required	≥1
WHO, 1980[3]	1-step	75	≥140 (7.8)	Not required	≥200 (11.1)	Not required	≥1
WHO,1999[4]	1-step	75	≥126 (7.0)	Not required	140 (7.8	Not required	≥1
American Diabetes Association[5]	1-step<br>or<br>2-step**	75<br><br>100	≥95 (5.3)<br><br>≥95 (5.3)	≥180 (10.0)<br><br>≥180 (10.0)	≥155 (8.6)<br><br>≥155 (8.6)	Not required<br><br>≥140 (7.8)	≥2<br><br>≥2
Australasian Diabetes in Pregnancy Society[6]	1-step	75	99 (5.5)	Not required	144 (8.0)	Not required	≥1
IADPSG, 2010[7]	1-step	75	≥92 (5.1)	≥180 (10.0)	≥153 (8.5)	Not required	≥1

Adapted from Table 2.1 (Lindsay)[8] and Table 1 (Donovan et al.)[9]

WHO, World Health Organization; IADPSG, International Association of the Diabetes and Pregnancy Study Groups

*Step one of the two-step approach is a 50 g glucose challenge test with a cutoff of ≥130-140 mg/dL (7.2-7.8 mmol/L), depending on the institution. Plasma glucose levels less than the cutoff are considered GDM-negative, and no further testing is required.[10]

**One-step approach may be preferred in clinics with a high prevalence of GDM.

References

1. Carpenter, M.W. and D.R. Coustan, *Criteria for screening tests for gestational diabetes.* Am J Obstet Gynecol, 1982. **144**(7): p. 768-73.
2. Kleinwechter, H., et al., *Gestational diabetes mellitus (GDM) diagnosis, therapy and follow-up care: Practice Guideline of the German Diabetes Association(DDG) and the German Association for Gynaecologyand Obstetrics (DGGG).* Exp Clin Endocrinol Diabetes, 2014. **122**(7): p. 395-405.
3. *WHO Expert Committee on Diabetes Mellitus*, in *World Health Organisation Technical Report Series 646*. WHO Expert Committee. 1980, World Health Organization: Geneva.
4. *Diagnostic criteria and classification of hyperglycaemia first detected in pregnancy*, in *WHO/NMH/MND/13.2*. 2013, World Health Organization: Geneva.
5. American Diabetes, A., *Diagnosis and Classification of Diabetes Mellitus.* Diabetes Care, 2010. **33**(Suppl 1): p. S62-S69.
6. Hoffman, L., et al., *Gestational diabetes mellitus--management guidelines. The Australasian Diabetes in Pregnancy Society.* Med J Aust, 1998. **169**(2): p. 93-7.
7. *International Association of Diabetes and Pregnancy Study Groups Recommendations on the Diagnosis and Classification of Hyperglycemia in Pregnancy*. Diabetes Care, 2010. **33**(3): p. 676-682.
8. Lindsay, R., *What is gestational diabetes?*, in *Gestational diabetes: Origins, complications, and treatment*, C.J. Petry, Editor. 2014, Taylor & Francis Group, LLC: Boca Raton, FL. p. 33-47.
9. Donovan, L., et al., *Screening tests for gestational diabetes: A systematic review for the u.s. preventive services task force.* Annals of Internal Medicine, 2013. **159**(2): p. 115-122.
10. VanDorsten, J.P., et al., *National Institutes of Health Consensus Development Conference statement: Diagnosing gestational diabetes mellitus.* NIH Consens State Sci Statements, 2013. **29**(1): p. 1-30.
