# Supplementary figures and images for: First trimester prenatal screening biomarkers and gestational diabetes mellitus: A systematic review and meta-analysis
Source: PLoS One. 2018 Jul 26;13(7):e0201319. doi: 10.1371/journal.pone.0201319 (PMC6062092; doi:10.1371/journal.pone.0201319)

**S1 Fig.** Funnel plot of the studies reporting on PAPP-A MoM levels among women with and without GDM.

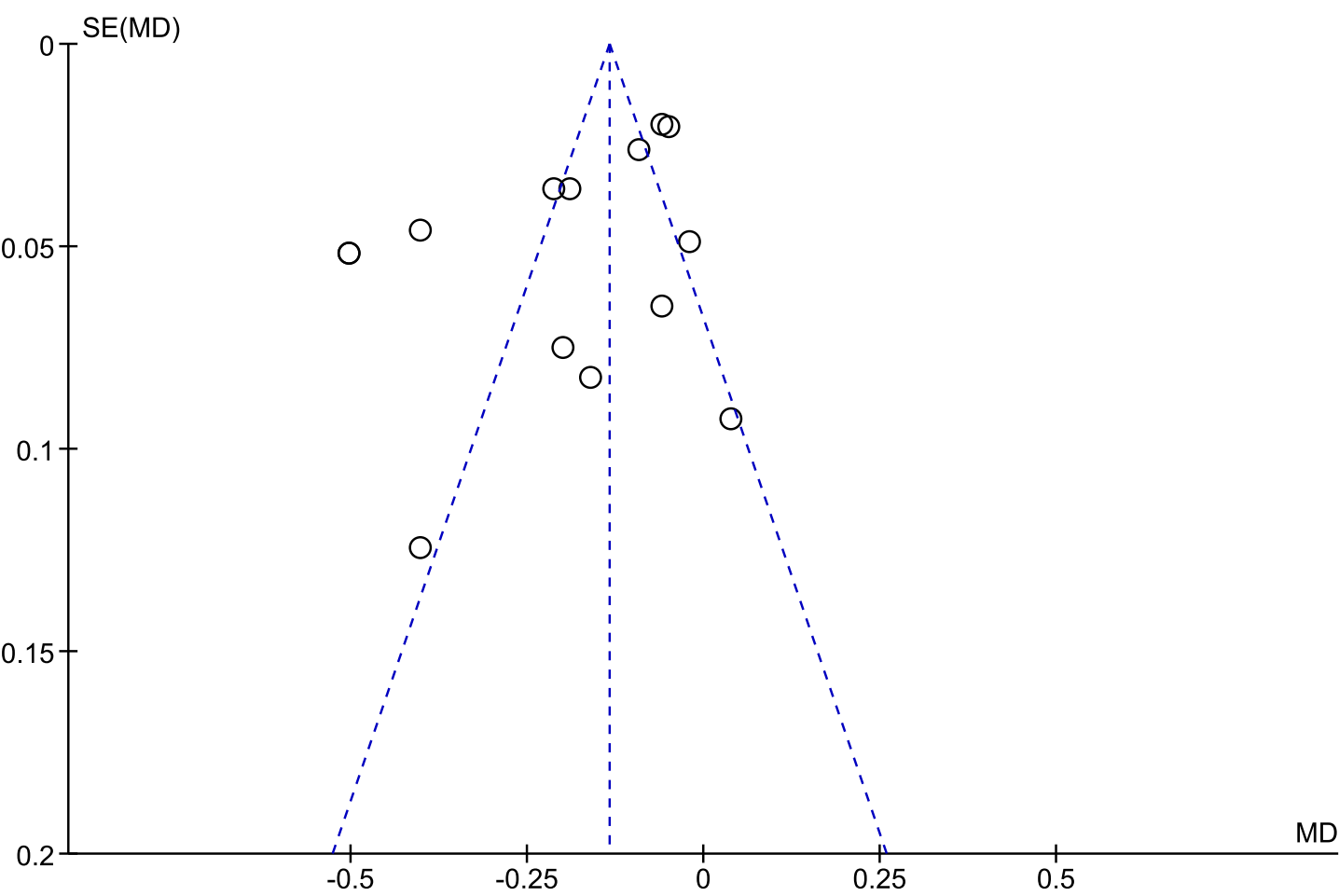

Supplement: S1 Fig — (PDF) [file pone.0201319.s005.pdf]
