# Supplementary material for: First trimester prenatal screening biomarkers and gestational diabetes mellitus: A systematic review and meta-analysis
Source: PLoS One. 2018 Jul 26;13(7):e0201319. doi: 10.1371/journal.pone.0201319 (PMC6062092; doi:10.1371/journal.pone.0201319)

S3 Fig. Forest plot of the studies reporting on PAPP-A MoM levels among women with and without GDM stratified by biomarker assay method.

Study or Subgroup	GDM Mean	GDM SD	GDM Total	Control Mean	Control SD	Control Total	Weight	Mean Difference IV, Random, 95% CI
**1.3.1 Delfia Xpress**								
Beneventi 2011	0.7	0.52	228	1.2	0.59	228	7.5%	-0.50 [-0.60, -0.40]
Savvidou 2012	0.94	0.55	779	1	0.55	41007	8.6%	-0.06 [-0.10, -0.02]
Lovati 2013	0.9	0.6	307	1.3	0.6	366	7.7%	-0.40 [-0.49, -0.31]
Beneventi 2014	1.06	0.59	112	1.22	0.64	112	6.1%	-0.16 [-0.32, 0.00]
Syngelaki 2015	0.95	0.57	787	1	0.89	30438	8.6%	-0.05 [-0.09, -0.01]
Cheuk 2016- Early and Late GDM Groups Combined	0.97	0.5	169	0.99	0.57	351	7.6%	-0.02 [-0.12, 0.08]
Xiao 2017	0.88	0.5	599	0.97	0.52	986	8.4%	-0.09 [-0.14, -0.04]
**Subtotal (95% CI)**			**2981**			**73488**	**54.5%**	**-0.18 [-0.28, -0.07]**
Heterogeneity: Tau² = 0.02; Chi² = 113.66, df = 6 (P < 0.00001); I² = 95%								
Test for overall effect: Z = 3.28 (P = 0.001)								
**1.3.2 BRAHMS Kryptor**								
Ong 2000	0.85	0.57	49	1.05	0.67	4297	6.1%	-0.20 [-0.36, -0.04]
Husslein 2012	1.17	0.71	72	1.13	0.58	216	5.6%	0.04 [-0.14, 0.22]
Farina 2017	0.7	0.36	12	1.1	0.53	60	4.3%	-0.40 [-0.64, -0.16]
**Subtotal (95% CI)**			**133**			**4573**	**16.0%**	**-0.18 [-0.41, 0.05]**
Heterogeneity: Tau² = 0.03; Chi² = 8.62, df = 2 (P = 0.01); I² = 77%								
Test for overall effect: Z = 1.50 (P = 0.13)								
**1.3.3 Immulite 2000**								
Kulaksizoglu 2013	0.77	0.42	60	0.97	0.4	60	6.4%	-0.20 [-0.35, -0.05]
Wells 2015- Early GDM	0.94	0.5	63	1	0.53	1282	6.9%	-0.06 [-0.19, 0.07]
Wells 2015- Late GDM	0.79	0.57	301	1	0.53	1282	8.1%	-0.21 [-0.28, -0.14]
Sweeting 2017- Early and Late GDM Groups Combined	0.81	0.46	248	1	0.56	732	8.1%	-0.19 [-0.26, -0.12]
**Subtotal (95% CI)**			**672**			**3356**	**29.5%**	**-0.18 [-0.23, -0.12]**
Heterogeneity: Tau² = 0.00; Chi² = 4.26, df = 3 (P = 0.23); I² = 30%								
Test for overall effect: Z = 6.25 (P < 0.00001)								
**Total (95% CI)**			**3786**			**81417**	**100.0%**	**-0.17 [-0.24, -0.10]**
Heterogeneity: Tau² = 0.01; Chi² = 136.59, df = 13 (P < 0.00001); I² = 90%								
Test for overall effect: Z = 4.79 (P < 0.00001)								
Test for subgroup differences: Chi² = 0.00, df = 2 (P = 1.00), I² = 0%								

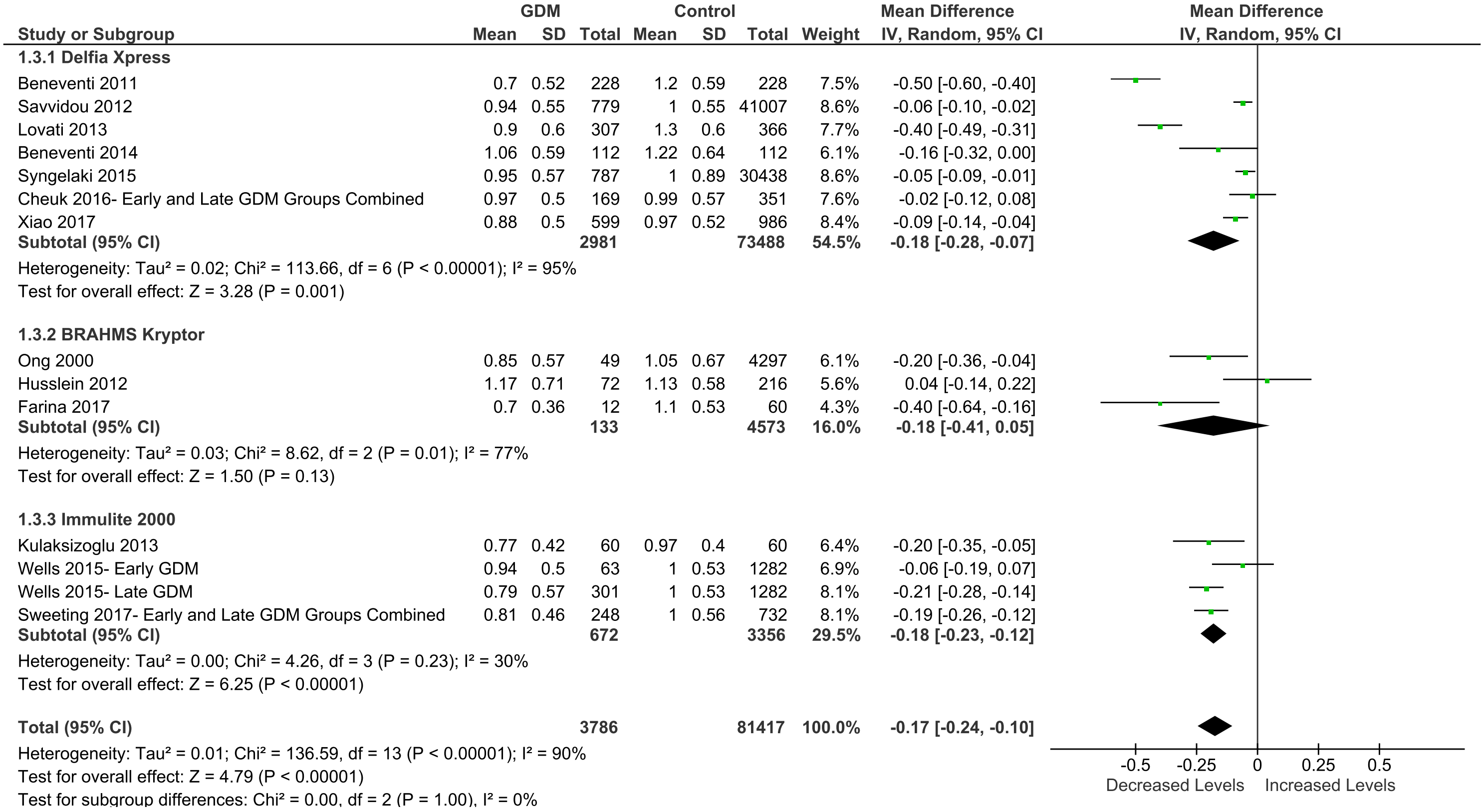

Supplement: S3 Fig — (PDF) [file pone.0201319.s007.pdf]
