# Supplementary material for: First trimester prenatal screening biomarkers and gestational diabetes mellitus: A systematic review and meta-analysis
Source: PLoS One. 2018 Jul 26;13(7):e0201319. doi: 10.1371/journal.pone.0201319 (PMC6062092; doi:10.1371/journal.pone.0201319)

S4 Fig. Forest plot of the studies reporting on PAPP-A MoM levels among women with and without GDM removing large studies.

	GDM			Control				Mean Difference
**Study or Subgroup**	**Mean**	**SD**	**Total**	**Mean**	**SD**	**Total**	**Weight**	**IV, Random, 95% CI**
Ong 2000	0.85	0.57	49	1.05	0.67	4297	7.5%	-0.20 [-0.36, -0.04]
Beneventi 2011	0.7	0.52	228	1.2	0.59	228	8.9%	-0.50 [-0.60, -0.40]
Husslein 2012	1.17	0.71	72	1.13	0.58	216	7.1%	0.04 [-0.14, 0.22]
Kulaksizoglu 2013	0.77	0.42	60	0.97	0.4	60	7.9%	-0.20 [-0.35, -0.05]
Lovati 2013	0.9	0.6	307	1.3	0.6	366	9.1%	-0.40 [-0.49, -0.31]
Beneventi 2014	1.06	0.59	112	1.22	0.64	112	7.5%	-0.16 [-0.32, 0.00]
Wells 2015- Early GDM	0.94	0.5	63	1	0.53	1282	8.4%	-0.06 [-0.19, 0.07]
Wells 2015- Late GDM	0.79	0.57	301	1	0.53	1282	9.5%	-0.21 [-0.28, -0.14]
Cheuk 2016- Early and Late GDM Groups Combined	0.97	0.5	169	0.99	0.57	351	9.0%	-0.02 [-0.12, 0.08]
Farina 2017	0.7	0.36	12	1.1	0.53	60	5.7%	-0.40 [-0.64, -0.16]
Sweeting 2017- Early and Late GDM Groups Combined	0.81	0.46	248	1	0.56	732	9.5%	-0.19 [-0.26, -0.12]
Xiao 2017	0.88	0.5	599	0.97	0.52	986	9.8%	-0.09 [-0.14, -0.04]
**Total (95% CI)**			**2220**			**9972**	**100.0%**	**-0.20 [-0.28, -0.11]**

Heterogeneity: Tau² = 0.02; Chi² = 95.67, df = 11 (P < 0.00001); I² = 89%
Test for overall effect: Z = 4.45 (P < 0.00001)

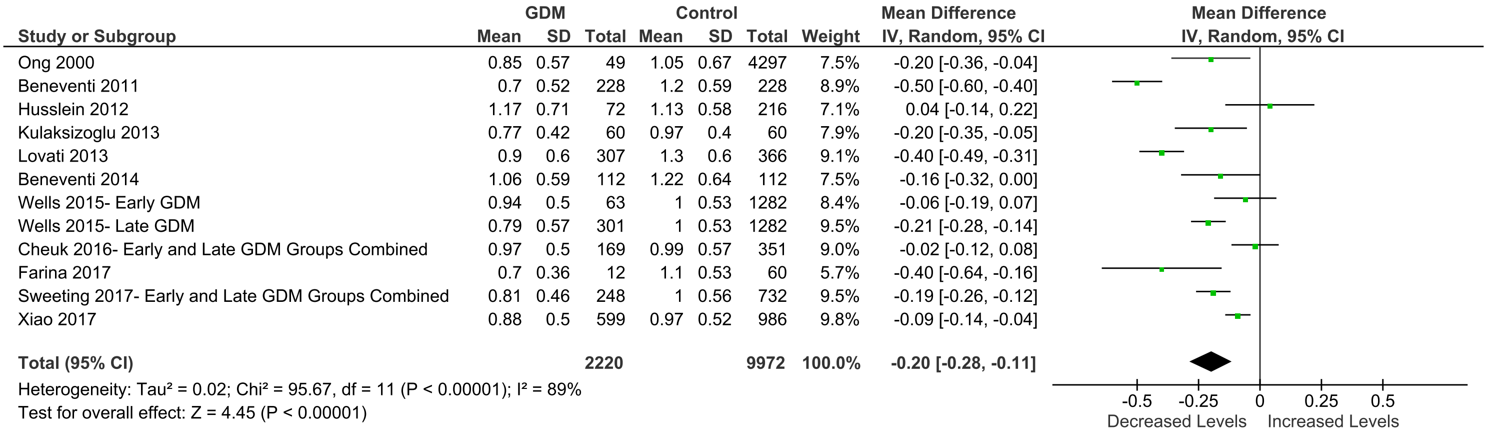

Supplement: S4 Fig — (PDF) [file pone.0201319.s008.pdf]
