# Supplementary material for: First trimester prenatal screening biomarkers and gestational diabetes mellitus: A systematic review and meta-analysis
Source: PLoS One. 2018 Jul 26;13(7):e0201319. doi: 10.1371/journal.pone.0201319 (PMC6062092; doi:10.1371/journal.pone.0201319)

S5 Fig. Forest plot of the studies reporting on PAPP-A MoM levels among women with and without GDM removing a study of lower quality.

	GDM			Control				Mean Difference
Study or Subgroup	Mean	SD	Total	Mean	SD	Total	Weight	IV, Random, 95% CI
Ong 2000	0.85	0.57	49	1.05	0.67	4297	6.5%	-0.20 [-0.36, -0.04]
Beneventi 2011	0.7	0.52	228	1.2	0.59	228	8.0%	-0.50 [-0.60, -0.40]
Husslein 2012	1.17	0.71	72	1.13	0.58	216	6.0%	0.04 [-0.14, 0.22]
Savvidou 2012	0.94	0.55	779	1	0.55	41007	9.2%	-0.06 [-0.10, -0.02]
Lovati 2013	0.9	0.6	307	1.3	0.6	366	8.2%	-0.40 [-0.49, -0.31]
Beneventi 2014	1.06	0.59	112	1.22	0.64	112	6.5%	-0.16 [-0.32, 0.00]
Syngelaki 2015	0.95	0.57	787	1	0.89	30438	9.1%	-0.05 [-0.09, -0.01]
Wells 2015- Early GDM	0.94	0.5	63	1	0.53	1282	7.4%	-0.06 [-0.19, 0.07]
Wells 2015- Late GDM	0.79	0.57	301	1	0.53	1282	8.7%	-0.21 [-0.28, -0.14]
Cheuk 2016- Early and Late GDM Groups Combined	0.97	0.5	169	0.99	0.57	351	8.1%	-0.02 [-0.12, 0.08]
Farina 2017	0.7	0.36	12	1.1	0.53	60	4.6%	-0.40 [-0.64, -0.16]
Sweeting 2017- Early and Late GDM Groups Combined	0.81	0.46	248	1	0.56	732	8.7%	-0.19 [-0.26, -0.12]
Xiao 2017	0.88	0.5	599	0.97	0.52	986	9.0%	-0.09 [-0.14, -0.04]
**Total (95% CI)**			**3726**			**81357**	**100.0%**	**-0.17 [-0.25, -0.10]**

Heterogeneity: Tau² = 0.02; Chi² = 135.48, df = 12 (P < 0.00001); I² = 91%

Test for overall effect: Z = 4.54 (P < 0.00001)

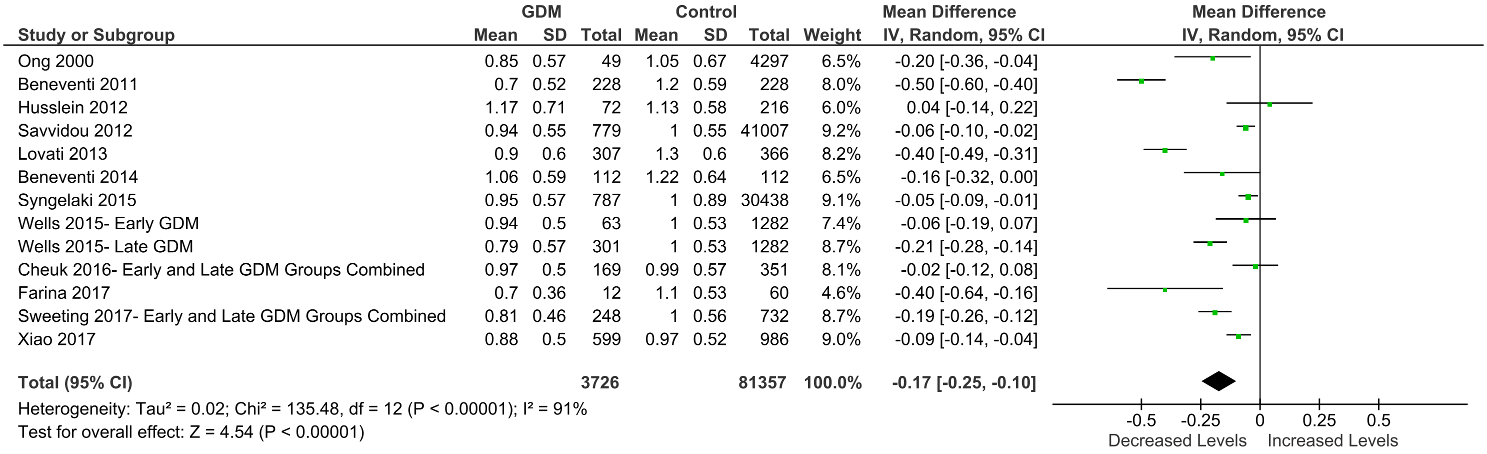

Supplement: S5 Fig — (PDF) [file pone.0201319.s009.pdf]
