# Supplementary material for: First trimester prenatal screening biomarkers and gestational diabetes mellitus: A systematic review and meta-analysis
Source: PLoS One. 2018 Jul 26;13(7):e0201319. doi: 10.1371/journal.pone.0201319 (PMC6062092; doi:10.1371/journal.pone.0201319)

S6 Fig. Forest plot of the studies reporting on PAPP-A MoM levels among women with and without GDM stratified by timing of GDM diagnosis.

	GDM			Control				Mean Difference
**Study or Subgroup**	**Mean**	**SD**	**Total**	**Mean**	**SD**	**Total**	**Weight**	**IV, Random, 95% CI**
**1.6.1 Late GDM Diagnosis**								
Ong 2000	0.85	0.57	49	1.05	0.67	4297	5.7%	-0.20 [-0.36, -0.04]
Beneventi 2011	0.7	0.52	228	1.2	0.59	228	7.1%	-0.50 [-0.60, -0.40]
Husslein 2012	1.17	0.71	72	1.13	0.58	216	5.3%	0.04 [-0.14, 0.22]
Savvidou 2012	0.94	0.55	779	1	0.55	41007	8.2%	-0.06 [-0.10, -0.02]
Kulaksizoglu 2013	0.77	0.42	60	0.97	0.4	60	6.0%	-0.20 [-0.35, -0.05]
Lovati 2013	0.9	0.6	307	1.3	0.6	366	7.3%	-0.40 [-0.49, -0.31]
Beneventi 2014	1.06	0.59	112	1.22	0.64	112	5.7%	-0.16 [-0.32, 0.00]
Syngelaki 2015	0.95	0.57	787	1	0.89	30438	8.1%	-0.05 [-0.09, -0.01]
Wells 2015- Late GDM	0.79	0.57	301	1	0.53	1282	7.7%	-0.21 [-0.28, -0.14]
Farina 2017	0.7	0.36	12	1.1	0.53	60	4.1%	-0.40 [-0.64, -0.16]
Sweeting 2017- Late GDM	0.83	0.5	138	1	0.56	732	7.3%	-0.17 [-0.26, -0.08]
Xiao 2017	0.88	0.5	599	0.97	0.52	986	8.0%	-0.09 [-0.14, -0.04]
**Subtotal (95% CI)**			**3444**			**79784**	**80.5%**	**-0.19 [-0.27, -0.11]**
Heterogeneity: Tau² = 0.02; Chi² = 128.73, df = 11 (P < 0.00001); I² = 91%								
Test for overall effect: Z = 4.78 (P < 0.00001)								
**1.6.2 Early GDM Diagnosis**								
Wells 2015- Early GDM	0.94	0.5	63	1	0.53	1282	6.5%	-0.06 [-0.19, 0.07]
Cheuk 2016- Early GDM	0.86	0.49	43	0.99	0.57	351	5.8%	-0.13 [-0.29, 0.03]
Sweeting 2017- Early GDM	0.79	0.41	89	1	0.56	732	7.2%	-0.21 [-0.30, -0.12]
**Subtotal (95% CI)**			**195**			**2365**	**19.5%**	**-0.14 [-0.24, -0.05]**
Heterogeneity: Tau² = 0.00; Chi² = 3.55, df = 2 (P = 0.17); I² = 44%								
Test for overall effect: Z = 2.93 (P = 0.003)								
**Total (95% CI)**			**3639**			**82149**	**100.0%**	**-0.18 [-0.25, -0.11]**
Heterogeneity: Tau² = 0.01; Chi² = 132.84, df = 14 (P < 0.00001); I² = 89%								
Test for overall effect: Z = 5.25 (P < 0.00001)								
Test for subgroup differences: Chi² = 0.68, df = 1 (P = 0.41), I² = 0%								

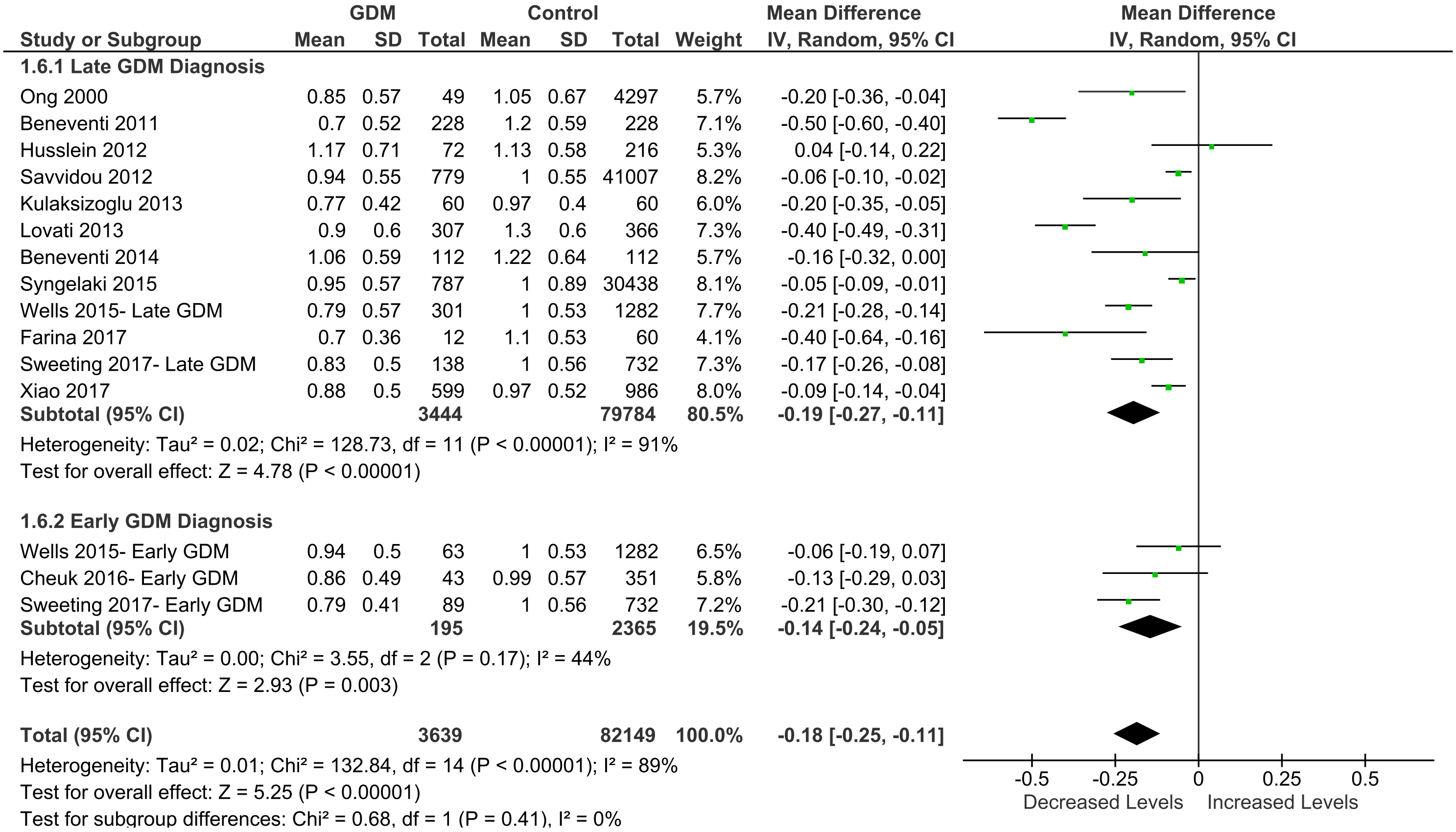

Supplement: S6 Fig — (PDF) [file pone.0201319.s010.pdf]
