# Supplementary material for: First trimester prenatal screening biomarkers and gestational diabetes mellitus: A systematic review and meta-analysis
Source: PLoS One. 2018 Jul 26;13(7):e0201319. doi: 10.1371/journal.pone.0201319 (PMC6062092; doi:10.1371/journal.pone.0201319)

S8 Fig. Forest plot of the studies reporting on free β-hCG MoM levels among women with and without GDM removing a large study**.**

	GDM			Control				Mean Difference
**Study or Subgroup**	**Mean**	**SD**	**Total**	**Mean**	**SD**	**Total**	**Weight**	**IV, Random, 95% CI**
Ong 2000	0.78	0.7	49	1.01	1	4297	3.7%	-0.23 [-0.43, -0.03]
Beneventi 2011	0.9	0.74	228	1	0.59	228	9.7%	-0.10 [-0.22, 0.02]
Husslein 2012	1.13	0.73	72	1.15	0.64	216	4.1%	-0.02 [-0.21, 0.17]
Kulaksizoglu 2013	0.93	0.53	60	0.97	0.29	60	6.3%	-0.04 [-0.19, 0.11]
Lovati 2013	1	0.67	307	1	0.67	366	14.2%	0.00 [-0.10, 0.10]
Cheuk 2016- Early and Late GDM Groups Combined	1.05	0.67	169	1.02	0.62	351	10.2%	0.03 [-0.09, 0.15]
Sweeting 2017- Early and Late GDM Groups Combined	0.98	0.6	248	0.99	0.64	732	19.0%	-0.01 [-0.10, 0.08]
Xiao 2017	1.01	0.66	599	1.06	0.66	986	32.7%	-0.05 [-0.12, 0.02]
**Total (95% CI)**			**1732**			**7236**	**100.0%**	**-0.04 [-0.08, 0.00]**

Heterogeneity: Tau² = 0.00; Chi² = 6.90, df = 7 (P = 0.44); I² = 0%
Test for overall effect: Z = 1.89 (P = 0.06)

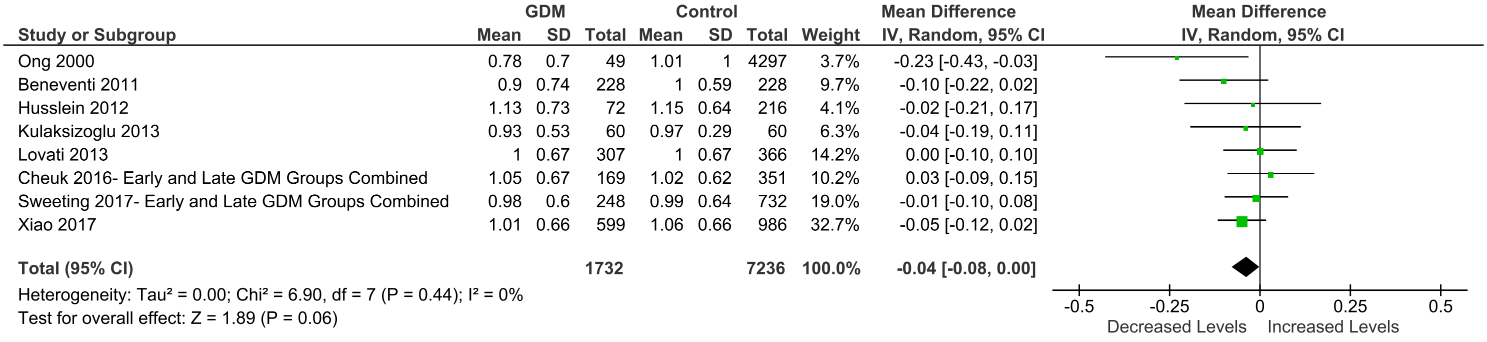

Supplement: S8 Fig — (PDF) [file pone.0201319.s012.pdf]
